# Machine Learning and Single-Cell Analysis Identify Molecular Features of IPF-Associated Fibroblast Subtypes and Their Implications on IPF Prognosis

**DOI:** 10.3390/ijms25010094

**Published:** 2023-12-20

**Authors:** Jiwei Hou, Yanru Yang, Xin Han

**Affiliations:** Department of Biochemistry and Molecular Biology, School of Medicine & Holistic Integrative Medicine, Jiangsu Collaborative Innovation Canter of Chinese Medicinal Resources Industrialization, Nanjing University of Chinese Medicine, Nanjing 210023, China; yrr@njucm.edu.cn

**Keywords:** idiopathic pulmonary fibrosis, fibroblast, bioinformatics, heterogeneity, predictive model

## Abstract

Idiopathic pulmonary fibrosis (IPF) is a devastating lung disease of unknown cause, and the involvement of fibroblasts in its pathogenesis is well recognized. However, a comprehensive understanding of fibroblasts’ heterogeneity, their molecular characteristics, and their clinical relevance in IPF is lacking. In this study, we aimed to systematically classify fibroblast populations, uncover the molecular and biological features of fibroblast subtypes in fibrotic lung tissue, and establish an IPF-associated, fibroblast-related predictive model for IPF. Herein, a meticulous analysis of scRNA-seq data obtained from lung tissues of both normal and IPF patients was conducted to identify fibroblast subpopulations in fibrotic lung tissues. In addition, hdWGCNA was utilized to identify co-expressed gene modules associated with IPF-related fibroblasts. Furthermore, we explored the prognostic utility of signature genes for these IPF-related fibroblast subtypes using a machine learning-based approach. Two predominant fibroblast subpopulations, termed IPF-related fibroblasts, were identified in fibrotic lung tissues. Additionally, we identified co-expressed gene modules that are closely associated with IPF-fibroblasts by utilizing hdWGCNA. We identified gene signatures that hold promise as prognostic markers in IPF. Moreover, we constructed a predictive model specifically focused on IPF-fibroblasts which can be utilized to assess disease prognosis in IPF patients. These findings have the potential to improve disease prediction and facilitate targeted interventions for patients with IPF.

## 1. Introduction

Idiopathic pulmonary fibrosis (IPF) is a parenchymal lung disease characterized by fibroblast proliferation and excessive accumulation of extracellular matrix (ECM) [1,2]. Unfortunately, the prognosis for patients with IPF is poor, with a median survival of approximately 3 years after diagnosis and limited treatment options available [3,4]. The underlying causes and mechanisms of fibrotic lung diseases, including IPF, are still not fully understood, and effective radical therapies are yet to be developed.

Numerous cell types, including alveolar epithelial cells, endothelial cells, immune cells, and fibroblasts, have been identified as contributors to fibrosis [2,5,6]. Among these, fibroblasts play a central role in the process of fibrogenesis, leading to the accumulation of extracellular matrix (ECM) and compromising lung structure and function [7]. In fibrotic lung tissue, fibroblasts demonstrate enhanced proliferative potential, increased migration, resistance to apoptosis, and invasive capacity, as well as leading to heightened deposition of ECM [8,9,10]. These characteristics significantly contribute to the pathogenesis of fibrosis, highlighting their potential value as both prognostic factors and therapeutic targets [11]. Pulmonary fibroblasts exhibit functional heterogeneity in lung homeostasis and disease [12]. Growing evidence suggests that specific subsets of fibroblasts actively contribute to lung pathophysiology by modulating the local immune microenvironment and producing ECM proteins [13,14,15]. The identification of these pathogenic fibroblast subsets presents new therapeutic possibilities for various fibrotic diseases.

In recent years, the application of single-cell RNA-sequencing (scRNA-seq) technology has significantly advanced our understanding of cellular heterogeneity in various pathological tissues [16,17]. Through this high-throughput analysis technique, the transcriptomic characteristics of fibroblasts in both normal and fibrotic lung tissues have been described [18]. However, there is still a lack of comprehensive understanding regarding the composition of fibroblast subsets, their gene expression profiling, and their specific functions in fibrotic lung tissue. In addition, the clinical association of fibroblast subtypes and their prognostic value for fibrogenesis remains to be illustrated. Here, we hypothesize that an integrated scRNA-seq analysis of lung fibroblasts can offer a more thorough characterization of fibroblast subtypes, novel insights into their biological characteristics, and signaling pathways they may activate in fibrotic lung tissue, which in turn may have an impact on clinical outcome.

Machine learning, a data analysis method that automatically constructs analytical models, has been widely utilized in clinical medicine [19]. Previous studies have demonstrated its potential in designing drugs, identifying pathologies, and developing predictive models [20,21,22]. In recent years, machine learning has been applied to diagnose and treat various diseases, including pulmonary fibrosis [23,24,25]. In these applications, least absolute shrinkage and selection operator (LASSO) logistic regression analysis, a linear regression method with regularization, is commonly utilized for high-dimensional analysis. Additionally, support vector machine–recursive feature elimination (SVM-RFE) can be employed to select optimal combinations of variables by leveraging its non-linear discrimination capabilities and ability to model different variable quantities [26]. Hence, the identification of biomarkers for idiopathic pulmonary fibrosis and the construction of prediction models using machine learning algorithms are of significant importance.

In the present study, we systematically classified fibroblast populations and revealed the molecular and biological characteristics of fibroblast subtypes in fibrotic lung tissue. Using hdWGCNA, we further identified the co-expressed gene modules associated with IPF-fibroblasts and addressed the valuable prognostic utility of signature genes for these IPF-related subtypes through a machine learning-based approach, providing valuable assistance for disease prediction and intervention.

## 2. Results

### 2.1. Single-Cell RNA Sequencing Reveals the Cellular Heterogeneity of Fibroblasts in Normal and Fibrotic Lung Tissues

**Figure 1:**
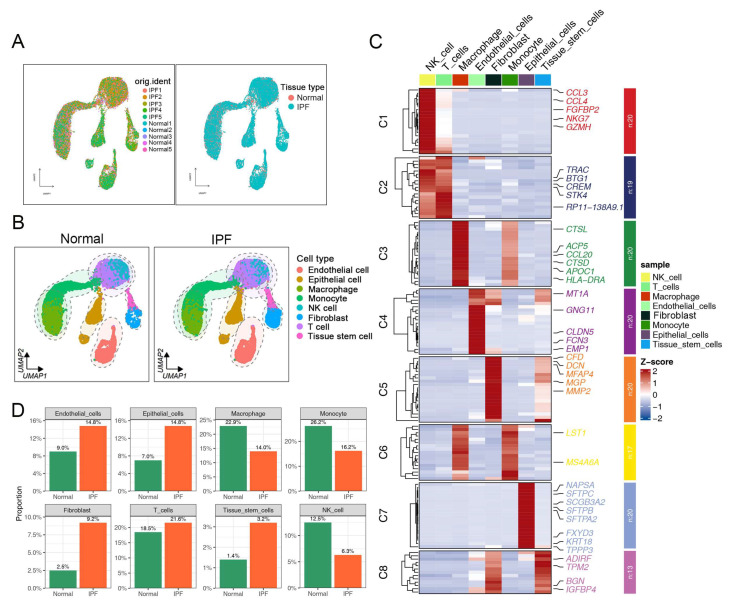
Integrated scRNA-seq analysis reveals heterogeneity of normal and fibrotic lung tissues, according to dataset GSE128033. (**A**) Cells on the UMAP plot of all 10 samples were colored as originating from normal and IPF patients. (**B**) Unbiased clustering of 26,129 cells reveals eight cellular clusters. Clusters are distinguished by different colors. (**C**) Heatmap showing representative differentially expressed genes between each cell population. (**D**) Cell proportions of eight cell types originating from normal and fibrotic lung tissues.

To explore the cellular composition and diversity of fibroblasts in both normal and fibrotic lung tissues, we collected and analyzed scRNA-seq data from patients with IPF. Specifically, we selected sequencing data from both normal and lower lobe samples, as pulmonary fibrosis often initiates in the lower lobe in clinical practice. Uniform manifold approximation and projection (UMAP) analyses identified eight major cell populations, including endothelial cells, epithelial cells, macrophages, monocytes, NK cells, fibroblasts, T cells, and tissue stem cells (Figure 1A,B). Figure 1C illustrates the expression of known lineage markers in the eight major cell clusters in both the normal and IPF groups. We next investigated the proportion of each cell population in the different sample sets (Figure 1D). As expected, we observed a significant increase in the fraction of the fibroblast cluster in the IPF group compared with the normal group.

**Figure 2:**
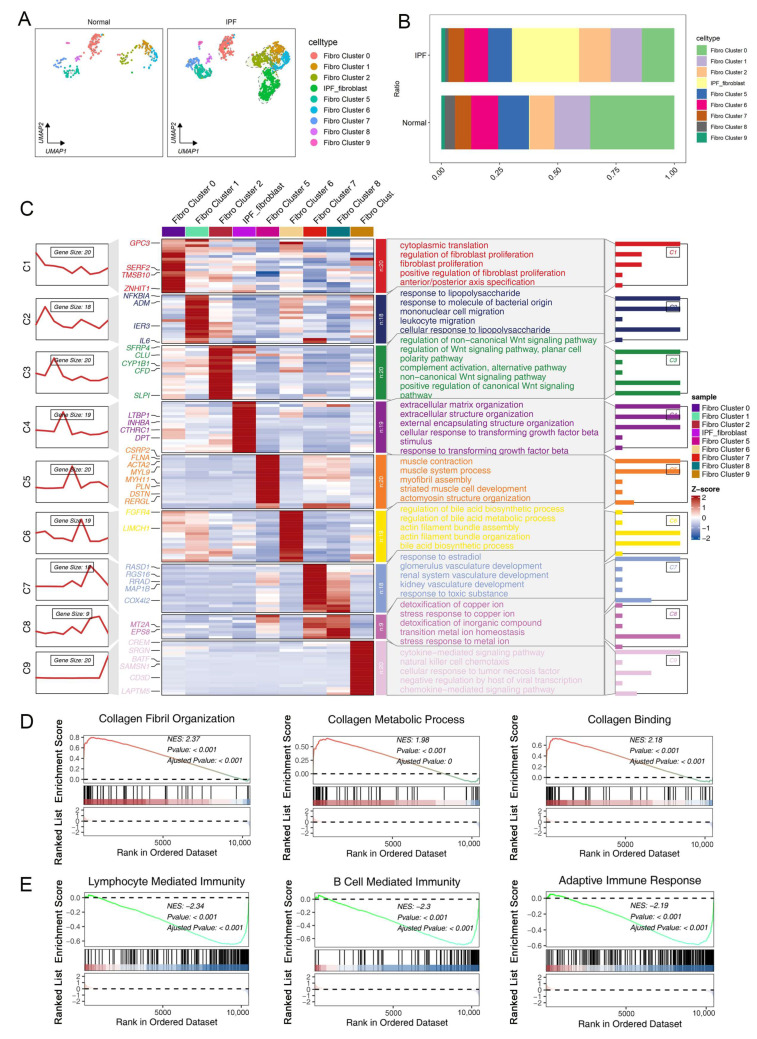
scRNA-seq analysis reveals heterogeneity of fibroblast subtypes in lung fibrosis. (**A**) Subclustering of fibrotic and normal lung fibroblasts further identified nine distinct subtypes. Each fibroblast subcluster is visualized via a color-coded UMAP plot. (**B**) Cell proportions of fibroblast subclusters in the lung tissues of normal and IPF patients. (**C**) Left panels: The series of diagrams illustrates the patterns of dynamic changes in representative differentially expressed genes (DEGs) in each fibroblast population. Middle panels: heatmap showing representative DEGs between each cell population. Right panels: representative enriched gene ontology (GO) terms for each cluster. (**D**,**E**) GSEA enrichment plots for representative signaling pathways upregulated (**D**) and downregulated (**E**) in IPF-related fibroblasts compared to other fibroblasts.

Then, we repeated the UMAP analysis to hierarchically cluster the fibroblasts. As shown in Figure S1, subclustering of fibrotic and normal lung fibroblasts further identified 10 distinct subtypes. We demonstrated that fibroblasts of cluster 4 and cluster 5 were significantly increased in fibrotic samples compared to normal lung samples, which were defined as IPF-fibroblast (Figure 2A,B). To more precisely characterize the distinctive patterns of differentially expressed gene signatures in these cell subtypes, a score was assigned to each gene based on its relative expression in each individual cell. These genes were then subjected to unsupervised clustering, resulting in the formation of distinct gene clusters (Figure 2C). In addition, we grouped genes with similar expression trends, resulting in the identification of nine distinct trends with implications in various biological functions, as revealed by the clustering results. It is noteworthy that genes in cluster 4, which exhibited high expression levels in IPF-fibroblasts, were found to be highly enriched for biological processes related to fibrogenesis, including extracellular matrix (ECM) organization, extracellular structure organization, and cellular response to transforming growth factor beta stimulus. (Figure 2C). Consistently, gene set enrichment analysis (GSEA) also suggested that IPF-fibroblasts expressed high levels of genes involved in the deposition of ECM, such as collagen fibril organization, collagen metabolic process, and collagen binding (Figure 2D). Moreover, the GSEA result showed a negative correlation between IPF-fibroblasts and pathways involving activation of immune response (Figure 2E). These findings suggest that IPF-fibroblasts play a critical role in the synthesis and production of extracellular matrix components within alveolar structures, indicating their potential culpability in the development and progression of IPF.

### 2.2. The Pseudotime Trajectory Analysis of Pathogenic Fibroblast Subtypes during Fibrogenesis

**Figure 3:**
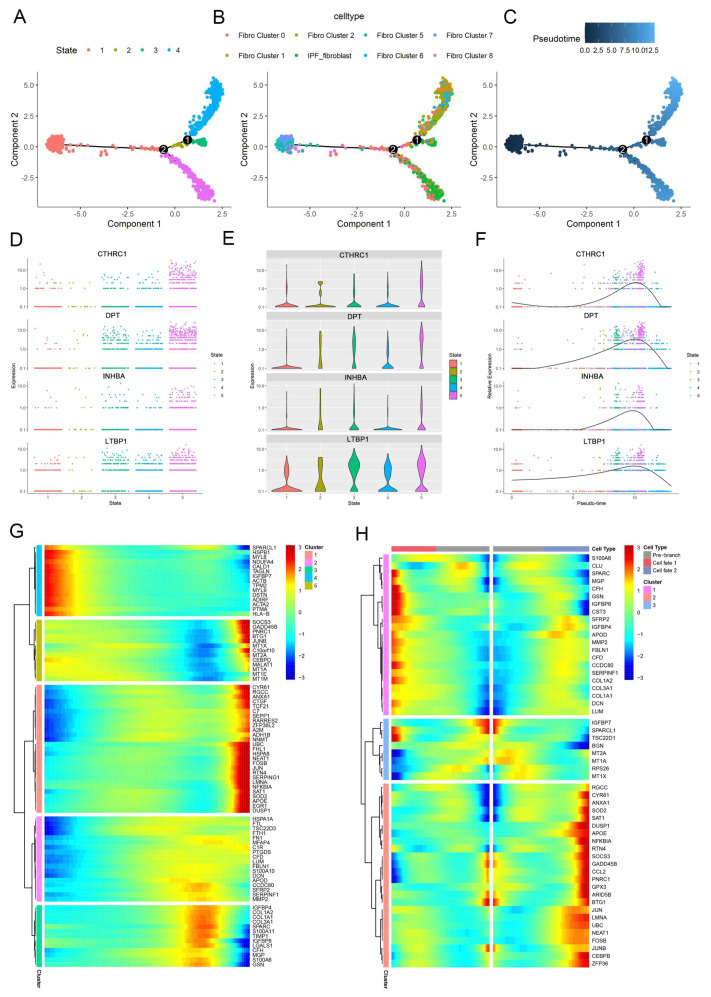
Trajectory analysis of fibroblast populations. (**A**–**C**) Monocle analysis for trajectory inference of the fibroblast subclusters, colored by cell cluster. The developmental trajectory of fibroblasts, color-coded by states (**A**), the associated cell subpopulations (**B**), and pseudotime (**C**). Scatter plots (**D**), violin plots (**E**), and pseudotime trajectories (**F**) show the expression of selected genes in different cell states as the pseudotime progresses. (**G**) A heatmap showing the dynamic changes in gene expression of the different cell clusters (**H**) The pseudotime heatmap shows the changes in selected genes after the changes in pseudotime.

To investigate the origins of IPF-fibroblasts in the development of IPF, pseudotime trajectory analysis of fibroblasts was further performed. Fibroblast clusters 5 and 7 were observed at the start of the trajectory, whereas IPF-fibroblasts were found at both ends of trajectory branches 1 and 2 (Figure 3A–C). We conducted further analysis of the dynamic expression changes of genes along the trajectory to identify genes that are critical for fibrogenesis. We identified several genes that exhibited the most significant changes in expression during pseudotime progression in IPF-fibroblasts, including collagen triple helix repeat-containing protein 1 (CTHRC1), dermatopontin (DPT), inhibitor beta A chain (INHBA), and latent-transforming growth factor beta-binding protein 1 (LTBP1) (Figure 3D–F). Furthermore, we conducted clustering of the top 100 genes based on their pseudotemporal expression patterns and subsequently analyzed the functional enrichments of each resulting cluster. As a result, we identified five distinct patterns of gene expression changes over pseudotime (Figure 3G). The genes assigned to cluster 2 exhibited high expression levels during the end stage and were primarily associated with extracellular matrix (ECM) organization. Conversely, genes assigned to cluster 4 displayed high expression levels during the beginning stage and were mainly enriched in processes related to mesenchymal migration and differentiation of glomerular mesangial cells (Figure S2A). Subsequently, we attempted to uncover the molecular mechanisms that distinguished the two branches. Our analysis of the gene expression dynamics revealed that, in conjunction with the fate 2 branch, the genes assigned to cluster 2 that were activated towards the end of the transition were primarily associated with the gene ontology (GO) terms “response to cytokine”, “negative regulation of apoptotic process”, and “cell proliferation”, all of which align with the characteristics of fibrotic differentiation (Figure S2B). Thus, these distinct gene expression patterns defined a successful IPF-fibroblast transition trajectory and highlighted a functional discrepancy within the pre-IPF-fibroblast subcluster.

### 2.3. Cell–Cell Communications Analyses in Lung Fibroblast Subpopulations

**Figure 4:**
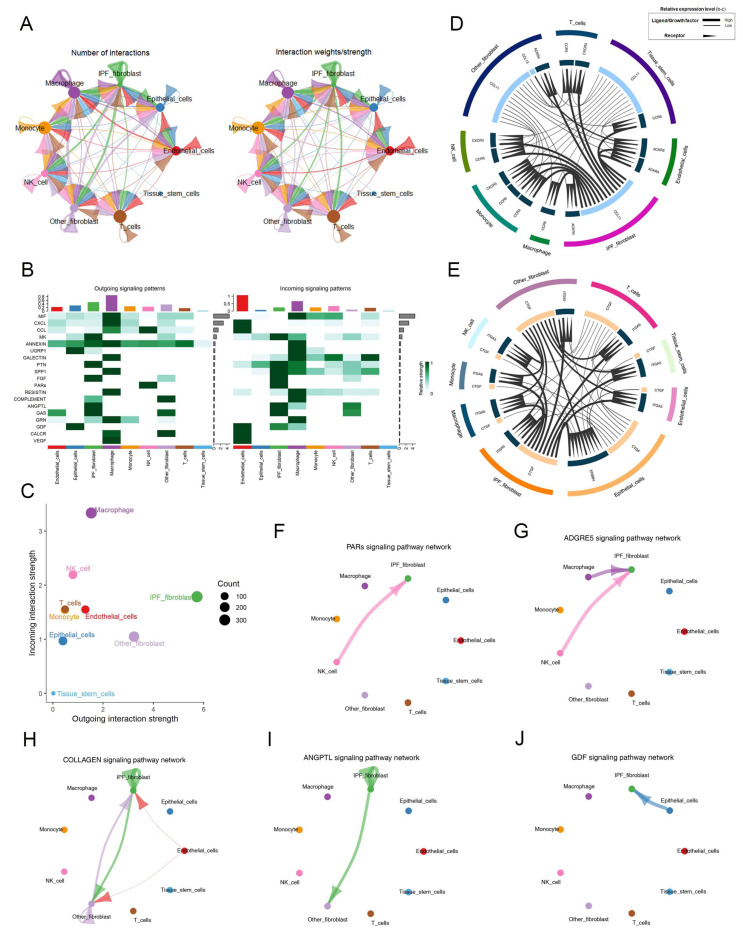
Cell communication analysis in fibroblast subpopulations. (**A**) Circle plots depict the number and strength of ligand–receptor interactions between pairs of cell populations. (**B**) The outgoing and incoming signaling patterns of fibroblasts and other cell populations. (**C**) A scatter plot reveals the variations in incoming and outgoing interaction strengths across all cell types. (**D**,**E**) Cell–cell ligand–receptor (LR) and cytokine-related pathway network in which fibroblasts interact with other cell populations in normal and fibrotic lung tissues. (**F**–**J**) Circle plots showing selected inferred differential signaling networks. The direction of the arrow indicates the direction of cell communication. The edge width represents the communication probability.

The availability of a single-cell dataset presented us with an exceptional opportunity to investigate cell–cell communication facilitated by ligand-receptor interactions. In order to elucidate the cell–cell communication network between fibroblast subpopulations and other cell types in fibrotic and normal lung tissues, we performed an analysis using CellChat, which was based on known ligand–receptor pairs and their cofactors [21]. Overall, IPF-fibroblasts exhibited strong communication abilities with other non-fibrotic cell types during the fibrogenesis process (Figure 4A). The results suggested that IPF-related fibroblasts exhibit a stronger secretory ability, as indicated by their higher levels of outgoing interaction strength compared to other fibroblasts (Figure 4B,C and Figure S3). Notably, our study revealed that IPF-fibroblasts are capable of directly interacting with other fibroblasts through the adhesive ligand–receptor pairs CCL11/ACKR4 and CTGF/ITGA5 (Figure 4D,E). We further demonstrated at the single-cell level that NK cells transmit PARs and ADGRE5-dependent signaling to IPF-fibroblasts (Figure 4F,G). Additionally, our CellChat analysis revealed an upregulation of pro-fibrosis signaling (such as COLLAGEN and ANGPTL) in the communication between IPF-fibroblasts and other fibroblasts, as well as an increase in GDF signaling in the communication between epithelial cells and IPF-fibroblasts (Figure 4H–J). Collectively, our findings suggest that IPF-fibroblasts and other cell types establish an interaction network that supports each other’s maintenance and function.

### 2.4. Identification of the Co-Expressed Gene Modules Associated with IPF-Fibroblasts by Using hdWGCNA 

**Figure 5:**
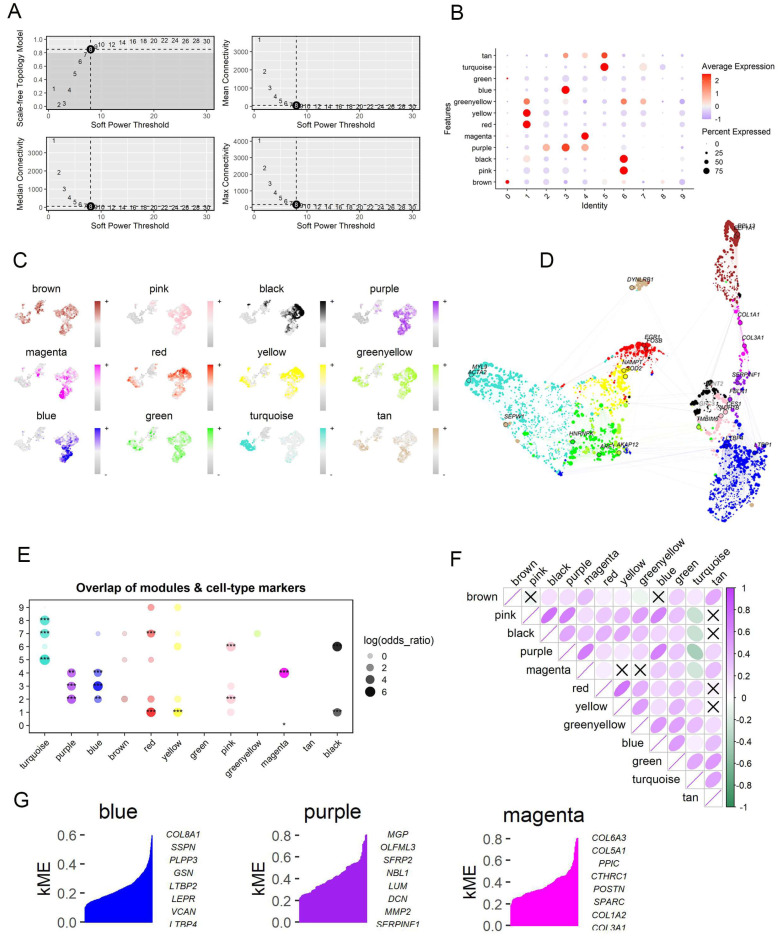
Identification of potential IPF-fibroblast-related genes associated with IPF by high-dimensional weighted gene co-expression network analysis (hdWGCNA). (**A**) The selection of soft-thresholding powers; left panels: the impact of soft-threshold power on the scale-free topology fit index; right panels: average network connectivity under different weighting coefficients. (**B**) Module activities in different fibroblast clusters. The hdWGCNA algorithm was used to estimate the module score. (**C**) UMAP plots as in Figure 2A, colored by MEs for the 12 co-expression gene modules. (**D**) An UMAP diagram Illustrating the co-expression network in fibroblasts. The edges show co-expression connections between genes and module hub genes, while each node represents a single gene. Point size is scaled by kME. Nodes are colored by co-expression module assignment. The top two hub genes per module are labeled. Network edges were downsampled for visual clarity. (**E**) Gene overlap within different modules (* *p* < 0.05, ** *p* < 0.01, *** *p* < 0.001). (**F**) The matrix plot visually represents the inter-module relationships by depicting the correlation between module eigengenes. (**G**) Three IPF-fibroblast-related gene modules were obtained, and the top hub gene was presented according to the hdWGCNA pipeline.

We utilized high-dimensional weighted gene co-expression network analysis (hdWGCNA), a comprehensive framework for co-expression network analysis in single-cell RNA sequencing data [22], to identify co-expressed gene modules and elucidate their functional roles within IPF-related fibroblasts. A scale-free co-expression network was established using an optimal soft thresholding power of 8 (Figure 5A). We identified a total of 12 distinct gene co-expression modules, among which the blue, purple, and magenta modules were highly activated, primarily in IPF-fibroblasts (clusters 3 and 4, as shown in Figure 5B,C). The correlation between each module was further investigated (Figure 5D–F). Figure 5G and Figure S4 presented the top 10 hub genes of the 12 modules and the protein-protein interaction (PPI) network of the identified hub genes in each module, respectively.

### 2.5. Machine Learning-Based Construction of the IPF-Fibroblast-Related Predictive Model for IPF 

Then, we focused on predicting the onset and progression of IPF using a predictive model based on hub genes from three IPF-fibroblast-related modules, which could differentiate IPF-related fibroblasts from other fibroblasts due to their gene significance. We utilized two bulk RNA-seq datasets for further analysis.

**Figure 6:**
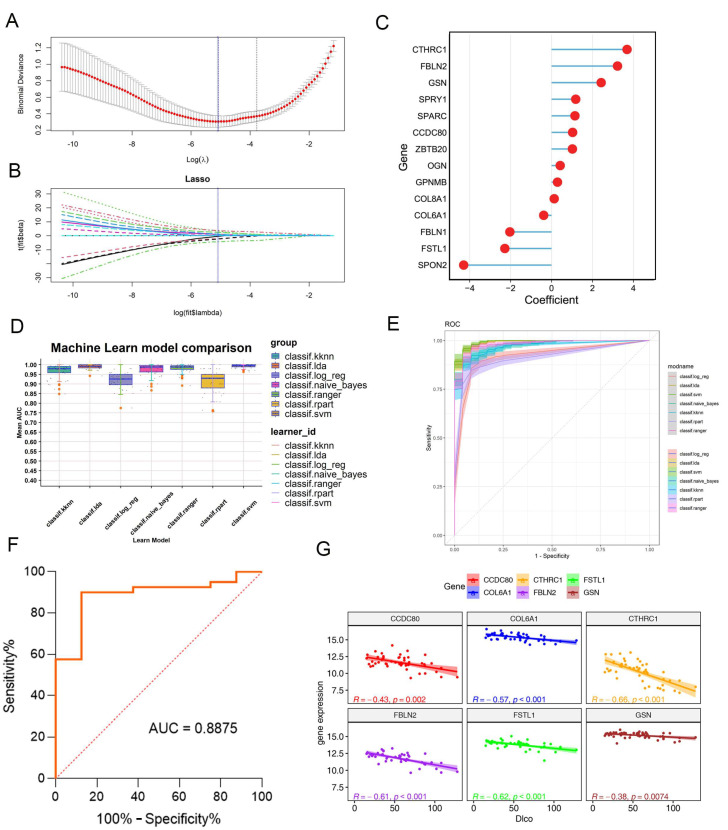
Construction of a machine learning model using bulk transcriptomic data. (**A**,**B**). LASSO regression was used to narrow down the IPF-fibroblast-related genes associated with IPF. (**A**) LASSO algorithm for selection features for IPF-fibroblasts. (**B**) Selection of genes with a non-zero coefficient for the construction of a model. (**C**) Coefficients of the identified genes within the prediction model. (**D**) Construction of the IPF-related fibroblast model through seven machine learning algorithms. (**E**) ROC values of all seven algorithms were shown in the training cohort. (**F**) ROC curve of the “svm” machine learning algorithm model in the validation cohort. (**G**) Correlation of IPF-fibroblast-related genes and diffusion lung capacity for CO (% DLCO) in control and IPF patients.

The GSE32537 dataset served as the training cohort, while the GSE14407 dataset was used to evaluate the predictive power of the final model developed. Initially, we conducted an analysis on the GSE32537 dataset, which consisted of 119 IPF patients. By employing the LASSO regression algorithm, we were able to identify 24 critical genes that exhibited a strong association with prognosis (Figure 6A,B). Subsequently, these identified genes underwent stepwise Cox proportional hazards regression, resulting in the final selection of 14 genes (Figure 6C). Next, we employed seven distinct machine learning algorithms and performed parameter optimization for each model using five repetitions of tenfold cross-validation. Subsequently, we evaluated the area under the curve (AUC) values of these models in the validation cohort. Through these rigorous mathematical procedures, we ultimately selected the “svm” machine learning algorithm model, which exhibited the highest AUC of 0.93 (Figure 6D,E). In addition, we constructed linear regression models to investigate the relationship between the expression levels of the 14 identified genes and lung function. Notably, Figure 6F demonstrates a significant negative correlation between the increased expression of CCDC80, COL6A1, CTHRC1, FBLN2, FSTL1, and GSN and a decline in the percent predicted diffusing capacity for lung carbon monoxide (% DLCO). The above results indicated the ideal predictive value of the IPF-fibroblast-related predictive model for IPF patients.

### 2.6. External Validation of an IPF-Fibroblast-Related Prognostic Signature 

**Figure 7:**
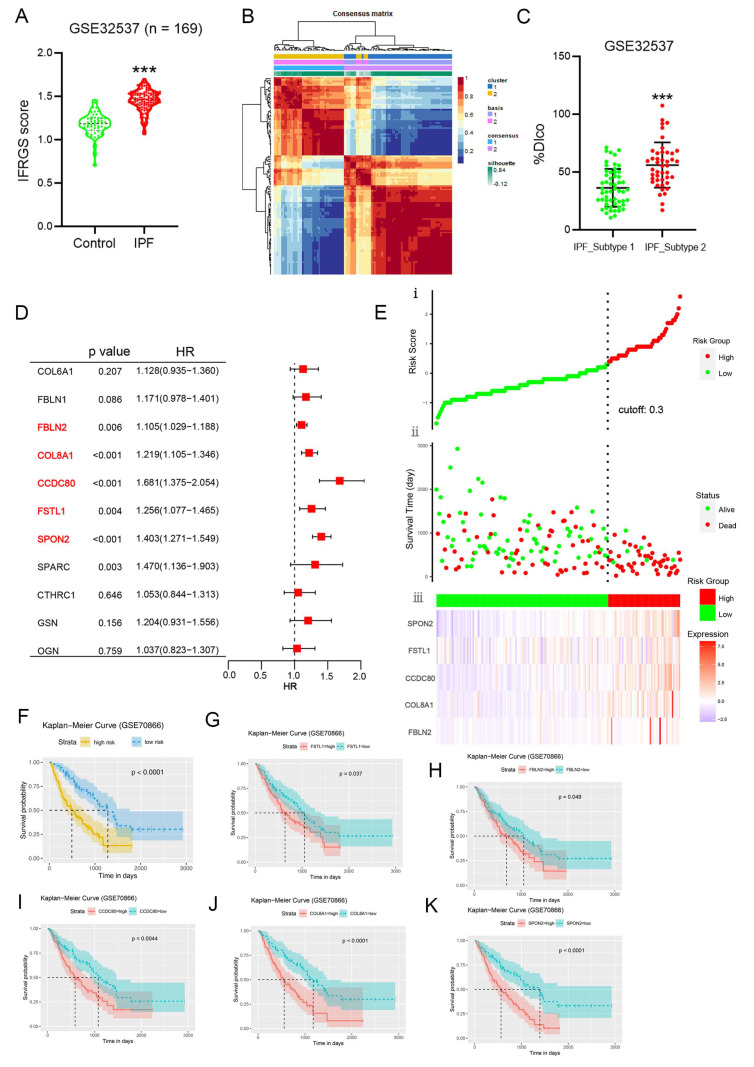
External validation of an IPF-fibroblast-related prognostic signature. (**A**) Boxplots depict the IPF-fibroblast-related genes (IFRGs) score levels in lung tissue of patients with IPF (*n* = 131) and normal controls (*n* = 39). Results are expressed as means ± SD (*** *p* < 0.001 vs. control). (**B**) A The heatmap of gene expression clusters for 131 IPF samples by unsupervised NMF illustrates two distinct expression patterns. (**C**) Box plots show the differences in diffusion lung capacity for CO (% DLCO) between two clusters (means ± SD; *** *p* < 0.001 vs. IPF_subtype 1). (**D**) Univariate Cox analysis of 11 IFRGs encoding secretory proteins associated with overall survival. (**E**) The risk score distribution, patient status, and mRNA expression heatmaps of the prognostic five-gene risk signature. (**F**) Kaplan–Meier curves for patients with high- or low-risk scores. (**G**–**K**) Kaplan–Meier survival analyses of IPF patients based on the expression of the identified genes.

To gain a deeper understanding of the correlation between the gene signature of IPF-fibroblasts and the process of fibrogenesis, we conducted an analysis of the IPF-fibroblast-related gene (IFRG) scores in both normal and fibrotic lung tissue. The results revealed a noteworthy increase in the IFRG scores within IPF lung tissue, indicating a potential association between the IFRGs and fibrogenesis (Figure 7A and Figure S5). We employed NMF clustering to classify IPF patients into two subtypes, namely, subtype 1 and subtype 2, using the clustering criteria derived from the differentially expressed IFRGs (Figure 7B). After comparing the two subtypes, we observed that patients with type 2 had a higher DLCO index, indicating better lung function in this group (Figure 7C). Given that fibroblasts associated with IPF may play a role in initiating and progressing the disease by releasing secretory proteins, we conducted a screening process using the IFRGs to identify these IPF-fibroblast-associated secretory proteins. Then, we evaluated the risk ratio for each secretory protein and identified five stable essential prognostic genes through multivariate Cox regression (Figure 7D). We displayed the distribution of risk scores based on pseudogenes, overall survival of IPF patients, and corresponding pseudogene expression profiles in another GEO dataset (GSE70866) to intuitively understand the prognostic effect of identified secretory protein-encoding genes. The results indicated that SPON2, FSTL1, CCDC80, COL8A1, and FBLN2 demonstrated high expressions in the high-risk group (Figure 7E). The patients in the high-risk group with the five-gene signature had a poor prognosis (Figure 7F). Furthermore, patients with IPF who exhibited elevated levels of these five gene expressions had shorter survival times (Figure 7J,K).

## 3. Discussion

Despite extensive research on human idiopathic pulmonary fibrosis (IPF), the underlying mechanisms responsible for the development of these diseases remain poorly understood. In addition, the available treatments for preventing or treating IPF are limited and often ineffective [27,28]. Fibrotic lung tissues comprise various cell subpopulations exhibiting diverse genetic and phenotypic traits. However, the precise mechanisms underlying the emergence of this heterogeneity during fibrosis development remain unclear. Herein, we conducted a thorough analysis of the fibroblast landscape in human idiopathic pulmonary fibrosis (IPF) and successfully identified two predominant subpopulations primarily found within fibrotic lung tissues, which have been designated as IPF-fibroblasts. Subsequently, we extensively investigated the distinctive characteristics and key regulatory pathways of distinct fibroblast subtypes. This in-depth exploration not only enhances our understanding of the pathogenesis of pulmonary fibrosis but also identifies potential targets for clinical therapies aimed at treating these conditions.

Previous single-cell RNA sequencing (scRNA-seq) studies that examined the heterogeneity of various lung-resident cell populations in pulmonary fibrosis have provided valuable insights but have only provided a limited snapshot of the overall landscape [29,30]. Through our extensive characterization of fibroblast heterogeneity, we have successfully identified a distinct fibroblast subtype, known as IPF-fibroblast, which exhibits a predominant presence in fibrotic lung tissue. We characterize the molecular features and identify novel markers of these fibroblast subpopulations related to IPF. We identify LTBP1, DPT, INHBA, and CTHRC1 as core enriched genes for IPF-fibroblasts in fibrotic lung tissues; however, their role in fibrogenesis remains largely elusive and requires further exploration. In addition, our findings revealed that IPF-fibroblasts exhibit elevated expression levels of genes associated with profibrotic processes, such as cellular response to transforming growth factor beta and collagen fibril organization [31,32]. Remarkably, our observations unveiled intriguing immunosuppressive characteristics in IPF-fibroblasts, implying their potential role in immunoregulation within the microenvironment of fibrotic pulmonary tissue. Consistent with this, previous studies have demonstrated that lung-resident fibroblasts play a crucial role in reshaping the local immune landscape, thereby facilitating the progression of the disease [14]. These scRNA-seq data reveal the dynamic molecular features of IPF-fibroblasts during development and provide a valuable resource for further studies.

The interactions between fibroblasts and other cell types in the lung are dynamic and multifaceted, playing a vital role in maintaining lung homeostasis and facilitating tissue repair processes. scRNA-seq analysis enables the identification of cell-surface receptors and their ligands that mediate the communication between fibroblasts and other cell types in the lung tissue. Through cell–cell communication analysis, it has been observed that IPF-fibroblasts have the potential to promote the differentiation of normal fibroblasts into fibrotic fibroblasts through CTGF/ITGA5 interaction [33]. In addition, we predicted that the PARs and GDF signaling pathways, which are mediated by NK cells and epithelial cells, respectively, would support the maintenance of the IPF-fibroblast phenotype. Furthermore, the trajectory study of IPF-fibroblasts changing from state 1 to state 2 further supports earlier findings and suggests that the majority of IPF-fibroblasts are probably derived from the activation of resident normal fibroblasts [7]. These results suggest that the identified IPF-fibroblast subpopulation might have an important role in fibrogenesis and may serve as target cells for a fibrosis treatment.

In this work, high–dimensional weighted gene co–expression network analysis (hdWGCNA), which is an advanced bioinformatics strategy for cell-associated gene module detection [34], and LASSO analysis combined with univariate analysis were further applied to develop a prognostic IPF-fibroblast-related gene (IFRG) signature. One of the characteristics of the IPF-fibroblasts in fibrotic lung tissue is their high expression of secretory proteins such as CCDC80, CTHRC1, COL6A1, FBLN2, FSTL1, and GSN. Previous studies suggested that some of these proteins, such as FSTL1, increased and could promote epithelial–mesenchymal transition in the lung [35,36]. Gelsolin (GSN), a protein that severs and caps actin filaments and plays a pivotal role in regulating actin assembly, has been reported to be involved in fibroblast activation during the development of myocardial fibrosis [37]. While there is currently a lack of reported evidence linking GSN to IPF, our research indicates that it plays a significant role in the activation process of fibroblasts associated with IPF. Furthermore, in line with our research findings, studies have revealed that the specific subset of cells with elevated CTHCR1 expression within fibrotic lung tissue demonstrates the highest level of collagen expression [38]. Notably, our findings showed that the aforementioned proteins were inversely linked with the percent anticipated diffusing capacity for lung carbon monoxide (% DLCO), emphasizing the clinical importance of IFRGs in pulmonary fibrosis. Based on the above findings, we first employed seven machine learning algorithms to construct an IPF-fibroblast-related predictive model for IPF. Then, we applied cross-validation and ROC curve analysis to assess the model’s performance. After comparing the algorithms’ performance on the validation set, we finally selected the algorithm with the best performance as our final model. Recently, a study utilized machine learning to develop an IPF prediction model focused on the midkine gene [39]. Similar to our research, this study employed three different algorithmic models (SVM, Adaboost, and random forest) and determined, through ROC curve analysis, that the SVM algorithm exhibited the highest accuracy. These findings align with our own research, indicating that the SVM algorithm possesses a considerable advantage and accuracy in constructing predictive models.

Overall, the model can effectively predict the prognosis of IPF patients, providing valuable assistance for disease prediction and intervention.

Despite the importance of these data for advancing knowledge, we should acknowledge some limitations. First, despite our best efforts to ensure the robustness of our clustering analysis of fibroblasts in fibrotic lung tissue, larger datasets could further improve and refine our clustering results. Second, being computational and omic in nature, our work requires experimental validation of the IPF-fibroblast markers derived from our findings for identifying and characterizing fibroblast subtypes in fibrotic lung tissues.

## 4. Materials and Methods

### 4.1. Data Acquisition

In total, four independent public datasets were downloaded from the NCBI GEO databases (http://www.ncbi.nlm.nih.gov/geo/, accessed on 2 September 2023). Specifically, the single-cell RNA-seq dataset GSE128033 was utilized to analyze the heterogeneity of fibroblasts in normal and fibrotic lung tissues [40]. We selected the lower lobe samples from the dataset, including normal and fibrotic lung tissues, as pulmonary fibrosis often originates in the lower lobe in clinical practice [41]. Three bulk RNA-seq datasets (GSE32537, GSE110147, and GSE70866) were employed for the construction and validation of our predictive model. Essential information about the samples of the four given datasets is displayed in Table 1. For analyses of data from a public database, patient consent and ethics committee approval were not required.

### 4.2. scRNA-seq Data Processing

ScRNA-seq data processing was performed using the R ‘Seurat’ package (version: 4.3.0) as previously described [42]. Briefly, cells with gene expression levels below 300 genes or above 6500 genes, as well as those with mitochondrial gene expression exceeding 10%, were excluded, ensuring the inclusion of the majority of cells in the utilized datasets. The SCTransform function was then applied to normalize and scale raw counts, followed by principal component analysis (PCA). To mitigate batch effects across dissociated scRNA-seq raw data, the R ‘Harmony’ package (version: 0.1.1) was employed. Clustering analysis was performed based on the edge weights between any two cells, and a shared nearest-neighbor graph was generated using the Louvain algorithm, implemented in the FindNeighbors and FindClusters functions. The resulting cells were visualized using the uniform manifold approximation and projection (UMAP) algorithm. A similar procedure was applied for subclustering analysis. The Seurat “FindMarkers” function was used to identify preferentially expressed genes within clusters as well as differentially expressed genes (DEGs) between fibrotic- and normal-derived cells. Each cell cluster was subsequently annotated using known cell-type marker genes. The specific expression patterns of the identified genes at the single-cell level were visualized using the “scRNAtoolVis” package (version 0.0.5).

### 4.3. Trajectory and Cell–Cell Communication Analysis

Unsupervised pseudotemporal analysis was conducted using the “Monocle” package (version 2.26.0) with the DDR-Tree algorithm and default parameters to investigate the trajectory of fibroblasts. Subsequently, the ‘plot_pseudotime_heatmap’ function was utilized to generate a heatmap, visually representing the dynamic expression of module genes along the pseudotime trajectories of fibroblasts in fibrotic lung tissues. To identify potential interactions between and within fibroblasts and other cell populations, the “CellChat” package (version 1.6.1) was employed with the default settings of the recommended pipelines [43]. 

### 4.4. Enrichment Analysis 

The Seurat “FindMarkers” function was used to identify the DEGs of each cell subcluster. A fold change (|FC|) greater than 2 and an adjusted *p*-value less than 0.05 were used as the cut-off criteria. Based on the DEGs, the gene set enrichment analysis (ssGSEA) and gene ontology (GO) enrichment analyses between the cell subgroups were performed using the “clusterProfiler” package (version 4.7.1003). The functional enrichment result was shown using the “GseaVis” package (version 0.0.8).

### 4.5. High Dimensional Weighted Gene Co-Expression Network Analysis (hdWGCNA)

To identify potential IPF-fibroblast-related genes associated with IPF, we performed hdWGCNA by using the “hdWGCNA” package (version 0.1.1.9010) [34]. Briefly, metacells were constructed separately for each sample and each cell cluster with the hdWGCNA function MetacellsByGroups, aggregating 50 cells per metacell. For each cell population, we first subset the Seurat object for the cell population of interest and then performed the standard hdWGCNA pipeline by sequentially running the following functions with default parameters: TestSoftPowers, ConstructNetwork, ModuleEigengenes, ModuleConnectivity, and RunModuleUMAP.

### 4.6. Machine Learning-Based Construction of an IPF-Fibroblast-Related Predictive Model 

In order to assess the predictive potential of IPF-fibroblast-related genes (IFRGs) identified by hdWGCNA in the development of IPF, we collected two transcriptome sequencing datasets, namely, GSE32537 and GSE110147. These datasets were systematically collected and utilized as the training and testing cohorts, respectively. The identified IFRG signature was utilized to build a prediction model in the training set. This was accomplished by employing seven machine learning algorithms through the “mlr3” package (version 0.16.0). The machine learning algorithms included in our analysis are as follows: log_reg (logistic regression), Ida (iterative dichotomizer 3), ranger (random forest), SVM (support vector machines), nave_bayes (naive Bayes classifier), rpart (recursive partitioning and regression trees), and kknn (k nearest neighbors). The model generation procedure was as follows: (a) Prognostic IFRGs (immune-related functional genes) were identified in the training cohort using least absolute shrinkage and selection operator (LASSO) and univariate Cox regression analyses [44]. (b) Seven machine learning algorithms were then applied to the prognostic IFRGs to create prediction models. Each algorithm was validated using 5-times-repeated tenfold cross-validation. (c) For each model, the “timeROC” R-package (version: 0.4) was utilized to generate ROC curves and evaluate the predictive capacity of the model. The model with the highest accuracy was selected as the final predictive model. (d) The predictive ability of the final model was evaluated using independent testing sets. 

### 4.7. Non–Negative Matrix Factorization (NMF) of IPF by IFRGs and Survival Analysis

To delve deeper into the subtypes of IPF, we employed the non-negative matrix factorization (NMF) algorithm from the “NMF” package (version 0.20.6) to gain additional insights. Firstly, we chose 30 IFRGs (identified by hdWGCNA) from the GSE32537 dataset. Following that, we employed the expression matrix of these selected genes in the NMF analysis to identify unique subtypes of IPF. Survival analysis of the identified secretory protein-encoding genes was conducted using the “survival” package (version 2.1.2). Survival status and risk scores of IPF patients in the high- and low-risk groups were analyzed through the “ggrisk” package (version 1.3). Statistical significance was set at *p* value < 0.05.

### 4.8. Statistical Analysis

All statistical analyses and data visualizations were performed using the R software (version 4.2.1). Pearson’s correlation coefficients were used to assess the correlations between two continuous variables. For quantitative data, either a two-tailed, unpaired Student *t*-test or a one-way analysis of variance (ANOVA) with Tukey’s multiple comparisons test was used to compare values between subgroups. *p* < 0.05 was considered to be statistically significant.

## 5. Conclusions

Overall, our study contributes to a better understanding of the heterogeneity within the fibroblast population in fibrotic lung tissue. The identification of distinct molecular and biological characteristics of fibroblast subtypes, along with the prognostic utility of signature genes, provides valuable insights into the pathogenesis of IPF. These findings have the potential to improve disease prediction and facilitate targeted interventions for patients with IPF.

## Figures and Tables

**Table 1:** Overview of the information of analyzed datasets.

Dataset	Year	Area	Species	Platform	Data Type	Number of Samples	
						Normal	IPF
GSE128033	2019	United States	Homo	GPL18573	scRNA-seq	10	8
GSE32537	2011	United States	Homo	GPL6244	Bulk RNA-seq	39	131
GSE110147	2018	Canada	Homo	GPL6244	Bulk RNA-seq	11	22
GSE70866	2015	Germany	Homo	GPL14550	Bulk RNA-seq	20	212

## Data Availability

The data that support this study are available within the article and its supplementary data files or available from the authors upon request.

## Supplementary Materials

The following supporting information can be downloaded at: https://www.mdpi.com/article/10.3390/ijms25010094/s1, Figure S1: Fibrotic and normal lung fibroblasts subcluster into distinct cell populations; Figure S2: Enrichment analyses of the gene clusters; Figure S3: Cell communication analysis in fibroblast subpopulations; Figure S4: High-dimensional weighted gene co–expression network analysis (hdWGCNA) reveals module-specific hub genes in IPF-related fibroblasts.
