# Supplementary material for: Machine Learning and Single-Cell Analysis Identify Molecular Features of IPF-Associated Fibroblast Subtypes and Their Implications on IPF Prognosis
Source: Int J Mol Sci. 2023 Dec 20;25(1):94. doi: 10.3390/ijms25010094 (PMC10778894; doi:10.3390/ijms25010094)
Supplement: Supplementary file 1 [file ijms-25-00094-s001.zip › ijms-2718404-supplementary.pdf]

Machine Learning and Single-Cell Analysis Identify Molecular Features of IPF-Associated Fibroblast Subtypes and Their Implications on IPF Prognosis

Jiwei Hou *, Yanru Yang and Xin Han *

Department of Biochemistry and Molecular Biology, School of Medicine & Holistic Integrative Medicine, Jiangsu Collaborative Innovation Canter of Chinese Medicinal Resources Industrialization, Nanjing University of Chinese Medicine, Nanjing 210023, China

* Correspondence: houjw@njucm.edu.cn (J.H.); xhan0220@njucm.edu.cn (X.H.)

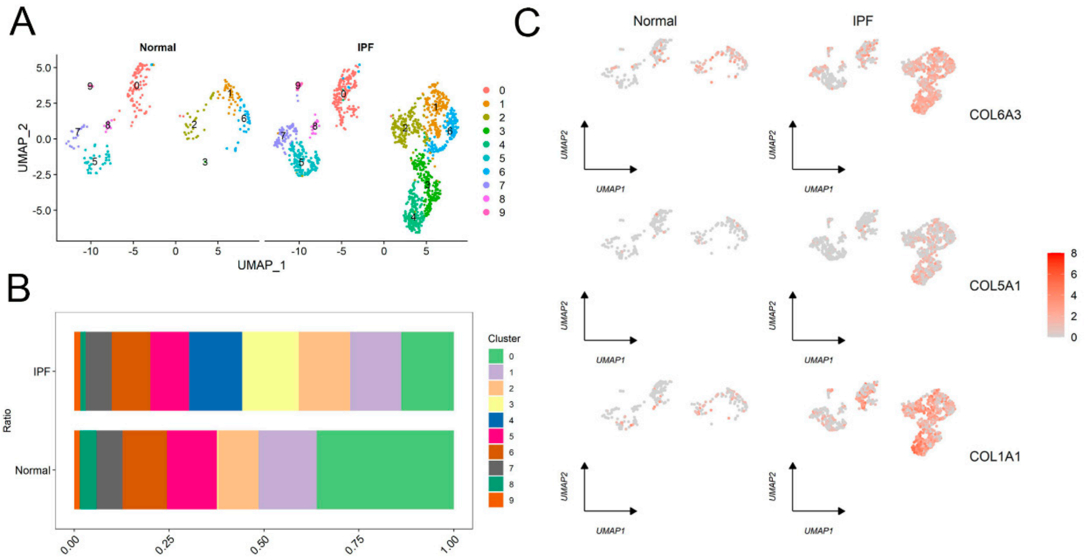

Figure S1： Fibrotic and normal lung fibroblasts subcluster into distinct cell populations. (A) Subclustering of fibrotic and normal lung fibroblasts further identified 10 distinct subtypes. Color-coded UMAP plot is shown and each fibroblast subcluster is defined on the right. (B) Cell proportions of fibroblast subclusters in fibrotic and normal lung tissues. Cells of cluster 4 and cluster 5 were significantly increased in fibrotic samples compared to normal scar samples. (C) Feature plots showing the expression of canonical markers for pulmonary fibrosis, in the fibroblast cell cluster.

A

	Enriched term	P value
Cluster 4	mesenchyme migration	0.0021
	glomerular mesangial cell differentiation	0.0021
	platelet aggregation	0.0025
Cluster 5	detoxification of copper ion	4.7073e-14
	stress response to copper ion	4.7073e-14
	detoxification of inorganic compound	7.0610e-14
Cluster 1	complement activation	0.0000014
	vasculature development	9.5748e-7
	cardiovascular system development	0.000001
	inflammatory response	0.000001
	innate immune response	2.9971e-7
	regulation of immune response	6.6659e-7
Cluster 2	extracellular matrix organization	1.1903e-9
	sequestering of iron ion	0.000016
	regulation of cell-substrate adhesion	0.0000019
	extracellular matrix organization	1.1903e-9
	extracellular structure organization	3.6451e-9
Cluster 3	collagen fibril organization	0.000005
	cellular response to amino acid stimulus	0.000013
	cellular response to amino acid stimulus	0.000013
	wound healing	8.0755e-7

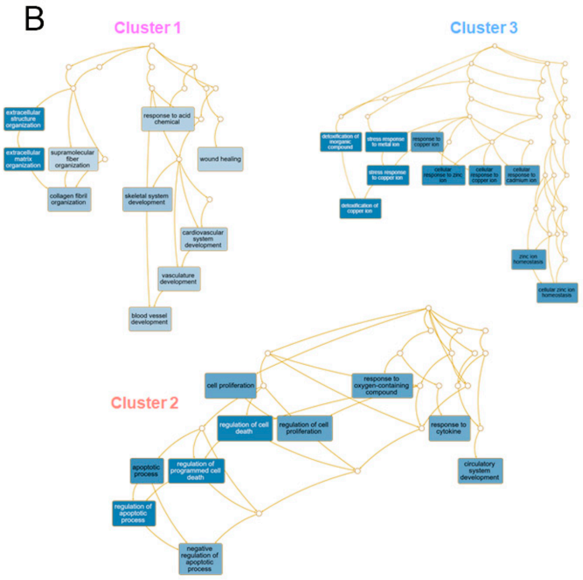

Figure S2： Enrichment analyses of the gene clusters. (A) The representative biological processes related to corresponding DEGs in Figure 3G. (B) GO terms enriched in the three gene clusters in Figure 3H.

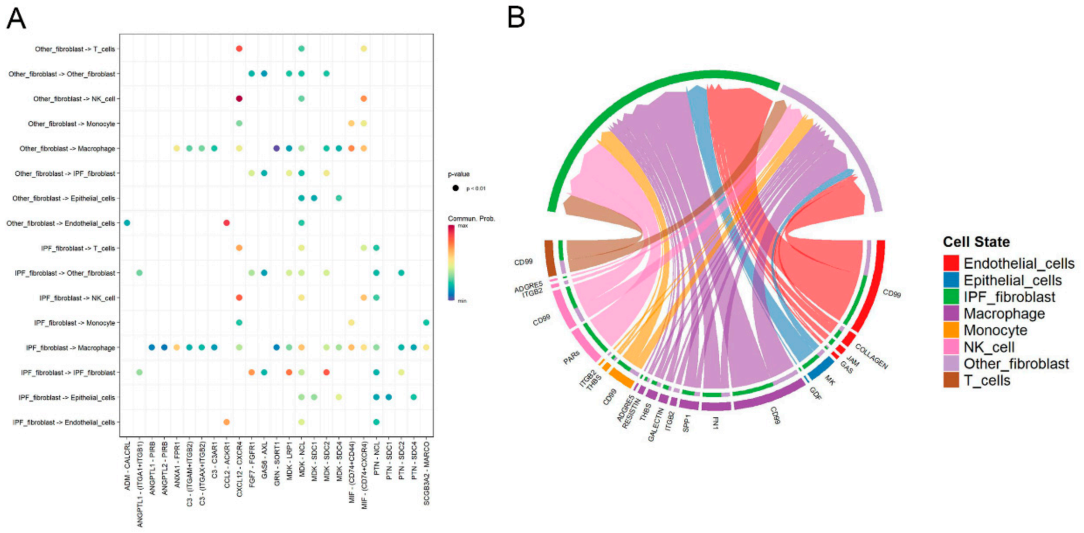

Figure S3:

Cell communication analysis in fibroblast subpopulations. (A) Cell–cell ligand-receptor (LRs) and cytokine-related pathway network in which fibroblasts interact with other cell populations in normal and fibrotic lung tissues. (B) Circle plots showing selected inferred differential signaling networks. The edge width represents the communication probability.

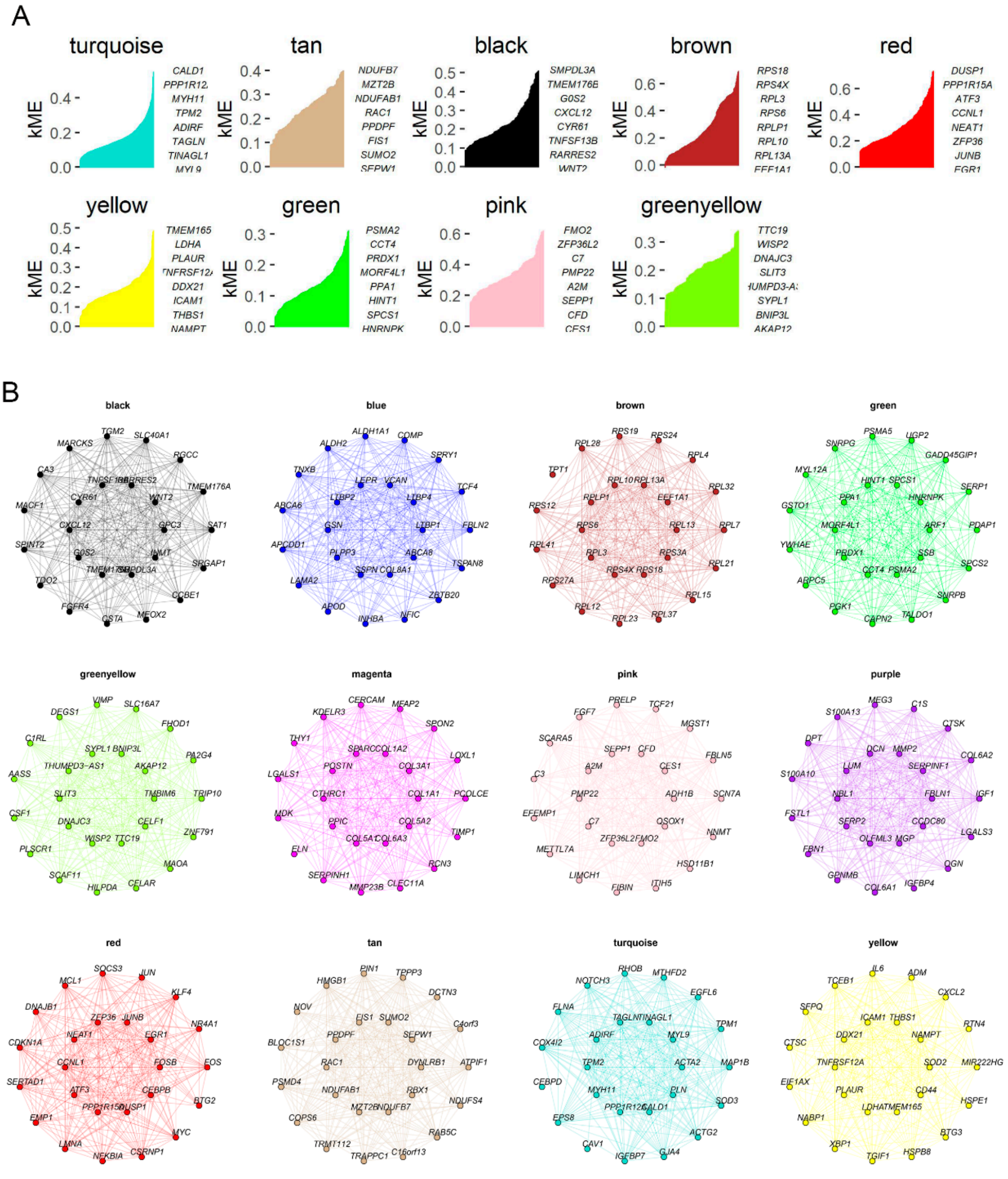

Figure S4:

High dimensional weighted gene co-expression network analysis (hdWGCNA) reveals module-specific hub genes in IPF-related fibroblasts. (A) kME (Eigengene-based connectivity) map showing the top 10 hub genes in each module ranked by kME across the macrophages. Genes with higher connectivity or kME values are considered more central or influential within their respective modules. (B) Protein–protein interaction (PPI) network of the identified hub genes in each module.

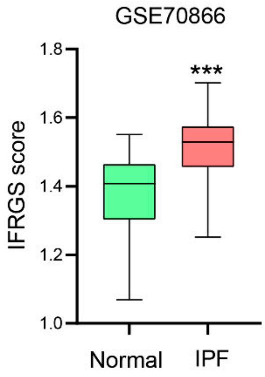

Figure S5

External validation of an IPF-fibroblast-related prognostic signature. (A) Boxplots depict the IPF-fibroblast-related genes (IFRGs) score levels in lung tissue of patients with IPF (n = 212) and normal controls (n = 20). Results are expressed as means ± SD (***$p < 0.001$ vs. Normal).
